# Allergen Peptides, Recombinant Allergens and Hypoallergens for Allergen-Specific Immunotherapy

**DOI:** 10.1007/s40521-013-0006-5

**Published:** 2014-02-26

**Authors:** Katharina Marth, Margarete Focke-Tejkl, Christian Lupinek, Rudolf Valenta, Verena Niederberger

**Affiliations:** 1 Division of Immunopathology, Department of Pathophysiology and Allergy Research, Center of Pathophysiology, Infectiology and Immunology, Medical University of Vienna, Vienna General Hospital, Vienna, Austria; 2 Department of Otorhinolaryngology, Medical University of Vienna, Vienna General Hospital, AKH 8J, 1090 Vienna, Austria

**Keywords:** Treatment, Allergen, Diagnosis, Allergy, Hypoallergens, IgE, Symptoms, Vaccines, Clinical efficacy, Recombinant allergen

## Abstract

Allergic diseases are among the most common health issues worldwide. Specific immunotherapy has remained the only disease-modifying treatment, but it is not effective in all patients and may cause side effects. Over the last 25 years, allergen molecules from most prevalent allergen sources have been isolated and produced as recombinant proteins. Not only are these molecules useful in improved allergy diagnosis, but they also have the potential to revolutionize the treatment of allergic disease by means of immunotherapy. Panels of unmodified recombinant allergens have already been shown to effectively replace natural allergen extracts in therapy. Through genetic engineering, several molecules have been designed with modified immunological properties. Hypoallergens have been produced that have reduced IgE binding capacity but retained T cell reactivity and T cell peptides which stimulate allergen-specific T cells, and these have already been investigated in clinical trials. New vaccines have been recently created with both reduced IgE and T cell reactivity but retained ability to induce protective allergen-specific IgG antibodies. The latter approach works by fusing *per se* non-IgE reactive peptides derived from IgE binding sites of the allergens to a virus protein, which acts as a carrier and provides the T-cell help necessary for immune stimulation and protective antibody production. In this review, we will highlight the different novel approaches for immunotherapy and will report on prior and ongoing clinical studies.

## Introduction

In industrialized societies, allergic diseases are among the most prevalent health issues, affecting up to 25 % of the population [1]. There are two main facets of the allergic response. The first is the immediate-type responses triggered by cross-linking of IgE antibodies on the surface of mast cells and basophil granulocytes by the allergen, followed by the release of inflammatory mediators, cytokines, and proteases, thus inducing symptoms within minutes of allergen contact. The second facet of the allergic response is caused by the activation of allergen-specific T cells, which produce pro-inflammatory cytokines and can lead to late-phase reactions and the more chronic forms of allergic inflammation. Although this phase of the allergic reaction can be significantly enhanced via IgE-facilitated allergen presentation by B cells [2], it can also occur without the presence of IgE epitopes. The latter has been shown by the induction of late asthmatic reaction after injection of short allergen peptides, which represented T cell epitopes but were devoid of IgE binding sites [3–5].

Although allergic symptoms can often be effectively suppressed using various drugs, only allergen immunotherapy is able to impact on the underlying immune mechanism and leads to long-lasting change in the course of allergic disease [6]. Conventional immunotherapy involves the repeated subcutaneous or sublingual administration of crude natural extracts of the allergen source over a period of two to three years or longer [7]. This treatment has been shown to improve symptoms and decrease the risk of progression of the disease to more severe forms (e.g., from rhinitis to asthma) [8, 9]. Allergen immunotherapy is effective for certain allergen sources (e.g., seasonal allergens, hymenoptera venom), while results are less convincing for others (e.g., mould spores, animal dander). Furthermore, treatment is time-consuming and somewhat arduous, with treatment outcomes that are unpredictable and vary from patient to patient and the potential for side effects.

Ideally, allergen extracts should contain stable and sufficient amounts of all relevant allergenic proteins. In reality, however, this is often not the case, as the content and quality of extracts is dependent on the source material, which shows natural variations, thus causing variability [10–13, 14•]. This issue remained unresolved until recombinant allergens became available [15, 16]. Over the last 25 years, a multitude of allergens have been produced as recombinant proteins and have been meticulously characterized. The grouping of homologous allergens into allergen families allowed the change from a taxonomy-based to a protein family-based classification [17–19], which elucidated the causes of cross-reactivity between taxonomically unrelated allergen sources [20, 21]. Furthermore, it was possible to distinguish between clinically relevant allergen molecules and others that were capable of binding IgE antibodies but are of minor clinical importance for allergic patients. This is the basis for the formulation of recombinant allergy vaccines that contain only molecules of clinical relevance for allergic patients [22].

Allergen components can be used in various ways to improve treatment options for allergic patients. Panels of unmodified recombinant or purified natural allergens, which emulate the allergen contents of natural extracts -- albeit in a highly standardized manner and lacking non-allergenic extract components -- have already been used in therapy for grass pollen- and birch pollen-allergic patients [23, 24]. A further developmental step was the use of recombinant technology to produce allergen derivatives, including hypoallergenic allergen derivatives [25–27], allergen hybrid proteins [28–30], and T cell peptides [31, 32], with the goal of improving the reliability and safety of vaccines and decreasing the number of injections.

In this article, we will describe the different strategies for the use of recombinant allergens and allergen derivatives for the treatment of allergic patients and provide an overview of studies that have been conducted or are currently ongoing. Additionally, we will discuss the monitoring of immunotherapy outcomes using new, test methods [33•], and discuss how the ever-growing availability of well-characterized allergen components may lead to personalized treatment of allergic patients. The last chapter of this article is devoted to the use of modified allergen components for the possible prophylactic treatment in individuals with a high risk of developing allergic disease.

## Treatment forms based on recombinant allergens and peptides

### Vaccines based on selected unmodified recombinant allergens

Several disadvantages of extract-based SIT (i.e., variable composition and allergen content [10–13, 14•], lack of allergen components [10, 11], contamination with other allergen sources [34], or bacterial components [35, 36]) can be overcome by using selected panels of unmodified recombinant or purified natural allergens for SIT. Evidence that this approach is effective has already been produced in two separate published clinical trials. In the first immunotherapy trial, grass pollen-allergic patients were treated with an equimolar mixture of the five major timothy grass pollen allergens (rPhl p 1, 2, 5a, 5b, and 6). A clinical benefit was associated with modification of the specific immune response with promotion of IgG and reduction of IgE antibodies [23]. In another trial, treatment of birch pollen-allergic patients with the recombinant major birch pollen allergen rBet v 1 was compared to treatment with purified natural Bet v 1 standard extract therapy or placebo. This multicenter study of 134 patients demonstrated that a single allergen was as effective as the purified natural allergen, which contained several isoforms, and the whole birch pollen extract [24]. Recombinant Bet v 1 has also been investigated for sublingual use [37, 38].

Because unmodified recombinant allergens have immunological features equal to their natural counterparts, they bear the risk of inducing adverse allergic events as a consequence of IgE or T cell reactivity. To overcome this problem, several different strategies have been developed to improve SIT vaccines.

### Vaccines with reduced IgE reactivity and retained T cell reactivity

Due to the fact that many IgE epitopes are conformational, IgE binding to allergens is often dependent on their correctly folded tertiary structure. One way to change or destroy conformational IgE epitopes is by fragmentation or oligomerisation of allergens. In a study performed in 1999--2000, a mixture of two recombinant Bet v 1 fragments and a recombinant Bet v 1 trimer were compared with placebo for subcutaneous SIT of birch pollen-allergic patients. Patients from both treatment groups developed protective IgG antibody responses against Bet v 1-related pollen and food allergens, and had a reduced boost of IgE memory responses during the birch pollen season. A reduction of cutaneous sensitivity to Bet v 1 was also observed [4, 25, 39, 40].

Another way to modify the structure of recombinant allergens is the production of folding variants by reduction and alkylation, as was demonstrated for Bet v 1 [41, 42]. In a dose-finding study using the resulting vaccine, individuals treated with the rBet v 1 folding variant exhibited lower symptom medication scores than the extract-treated control group [43].

Further attempts to reduce the IgE binding capacity of allergens have been made by the introduction of point mutations by site-directed mutagenesis of cysteines to disrupt disulfide bonds, the deletion of parts of the sequences, and the fusion of allergen variants. Thus far, these methods have not been used in patient therapy (reviewed in [15]).

### Vaccines based on T cell peptides

Synthetic T cell peptides may represent another alternative to recombinant allergens, as they encompass T cell epitopes while lacking the conformational IgE epitopes of the native allergen. When this approach was first investigated in cat-allergic patients, injection of short T cell peptides of Fel d 1, the major cat allergen, were associated with late-onset symptoms of rhinitis, asthma, and pruritus [44]. These issues were later largely overcome by the use of slightly longer peptides. However, the T cell-mediated side effects demonstrated that T cell-mediated late-phase reactions can occur in the absence of IgE epitopes. Current studies with immunodominant T cell epitopes showing promiscuous MHC binding are being conducted to define the optimal dose and dose interval [45•, 46].

A similar approach was used to vaccinate against the bee venom allergen phospholipase A2 (PLA2), which induced PLA2-specific IgG4, T cell hyporesponsiveness, Th1 cytokine deviation, and PLA2 peptide-specific IL-10 production, and which was well-tolerated by patients [47, 48]. The T cell peptide approach was further explored in a recent study using a combination of three long Bet v 1-derived peptides [49]. Due to the fact that rather long peptides were used and allergen-specific IgG responses were induced, this was not a purely T cell peptide-based approach, but was similar to an earlier study where recombinant Bet v 1 fragments were used to induce allergen-specific IgG [25, 50].

### Vaccines based on allergen derivatives with reduced IgE and T cell reactivity

A recently developed approach for a safe allergy vaccine is based on the hapten-carrier principle, where *per se* non-IgE-reactive allergen-derived peptides are covalently coupled to carrier proteins, such as viral proteins, in order to induce allergen-specific IgG antibodies with carrier-based T cell help [51]. Peptides, approximately 25--40 amino acids in length, derived from the IgE binding sites of allergens are derived from the IgE binding sites of allergens are selected and fused with a non-allergenic carrier protein that provides T cell help for the induction of carrier-specific and allergen-specific IgG.

This vaccine design was evaluated with peptides derived from the major allergens from timothy grass pollen (Phl p 1 and Phl p 5) [52, 53•], birch pollen (Bet v 1) [54], olive pollen (Ole e 1) [55], and the *Alternaria alternata* mould [56] by chemical coupling to keyhole limpet hemocyanin as carrier. Vaccination of mice and rabbits induced specific IgG antibodies that were able to inhibit the binding of IgE of allergic patients to the natural allergens.

As chemical conjugation may deliver end products of varying composition, it is not ideally suited for vaccine production under GMP conditions, it is not ideally suited for vaccine production. Therefore, defined recombinant fusion proteins consisting of the carrier protein and various numbers and combinations of the allergen-derived peptides were developed [57]. Examples of this approach are vaccines consisting of the rhinovirus-derived coat protein VP1 fused to Phl p 1-derived peptides [58] or vaccines based on the PreS domain of hepatitis B virus fused to cat-derived and birch pollen-derived peptides [59••, 60]. With the use of such technology, protective immunity against allergens and viral infections may be induced, thus providing the possibility to create combination vaccines for therapy and prophylaxis of allergy and infectious diseases [57].

Phase I and IIa clinical studies (NCT01350635, NCT01445002) were recently concluded on the BM32 vaccine, which consists of PreS fused to non-allergenic peptides from the 4 major timothy grass pollen allergens Phl p 1, 2, 5, and 6. A double-blind placebo-controlled multicenter phase IIb study (NCT01538979) is currently underway.

Another approach that was recently described involves the introduction of random mutations into allergen-encoding DNA and the expression of the recombinant mutants in phage libraries. IgE antibodies of allergic patients are used for phage enrichment to generate allergy vaccines with maintained structure but altered allergenic activity, as evaluated for Fel d 1. The resulting mutants induced blocking antibodies in immunized mice [61]. Table 1 provides an overview of the features of various allergen derivatives developed for immunotherapy. References with an asterisk refer to treatment forms which have been or currently are tested in SIT trials.

**Table 1:** Examples for recombinant allergens, allergen derivatives, and peptides developed for immunotherapy

	IgE reactivity	T cell reactivity	Induction of protective antibodies	Possible side effects:		References
				IgE	T cell	
				Mediated		
Recombinant wild-type allergens	+	+	+	+	+	[23*, 24*, 30, 37*, 62, 63]
Derivatives of recombinant allergens (mutants, fragments, oligomers)	+/-	+	+	-	+	[4*, 25*, 39*, 40*, 43*, 50, 61, 64–68, 69•, 70–72*]
T cell peptides	-	+	-	-	+	[3*, 32*, 45•*, 46–48*, 73*]
Peptide carrier fusion proteins	-	-	+	-	-	[52, 53•, 54–56, 58, 59••, 60, 74, 75]

*Indicates references which refer to clinical studies

### New routes for the application of vaccines based on recombinant allergens

Although subcutaneous and immunotherapy are currently the most frequently used routes of allergen delivery, other approaches such as oral, nasal, bronchial, epicutaneous, intraepithelial, intralymphatic, and rectal IT have been explored. Oral immunotherapy [76] has been proposed for desensitization in food allergy with the aim of inducing tolerance [77].

In this context, one new approach is edible transgenic rice containing storage protein bodies that are resistant to proteolytic degradation and may be suitable for bioencapsulation of allergens [64–66, 78]. In order to increase the safety of edible vaccines, hypoallergenic variants [67] and a Bet v 1 tolerogen generated by DNA shuffling [68] were introduced into transgenic rice. A possibility to improve sublingual or mucosal delivery of allergens is to combine recombinant allergens with mucoadhesive substances. This approach has thus far been evaluated in a murine model of chronic birch pollen respiratory allergy with rBet v 1 formulated in amylopectin-based microparticles [62].

Epicutaneous delivery of immunotherapy is another route currently under investigation for immunotherapy [79], and this involves either the use of patches [80, 81] or a specially developed delivery system [82] of allergen application. Both studies have provided evidence that epicutaneous immunotherapy may be safe and effective.

Transcutaneous immunotherapy via laser-generated micropores and co-application of CpG has been described to increase the safety of the therapy by abrogating the Th2 polarizing potential of skin immunization [83].

Another possibility for allergy vaccination is intralymphatic immunotherapy (ILIT). A study with a limited number of patients showed that ILIT with grass pollen or birch pollen extracts reduced nasal allergic symptoms without causing safety problems [84]. Using the same delivery method, a modular antigen transporter vaccine based on recombinant Fel d 1, the major cat allergen, was fused to the HIV TAT-derived translocation peptide and part of the human invariant chain in order to target the construct to the MHC class II pathway [69•]. In a first-in-human clinical study, ILIT with MAT-Fel d 1 stimulated regulatory T cell responses and increased cat dander-specific IgG levels [69•].

For cockroach allergy, a novel intranasal liposome-adjuvanted vaccine containing Per a 9 (*Periplaneta americana* arginine kinase) was tested in mice and found to attenuate allergic airway inflammation upon allergen re-exposure better than liposome-entrapped *P. americana* crude extract [85].

An approach based on rectal application used heat/phenol-inactivated *E. coli*-encapsulated, recombinant modified major peanut allergens and was tested in a phase I clinical trial. However, rectal administration resulted in frequent adverse reactions, including severe allergic reactions in 20 % of study participants [70].

## Companion diagnosis and monitoring of the success of SIT

Several mechanisms induced by the therapeutic injection of allergen extracts, such as the induction of blocking antibodies, have been suggested to mediate the beneficial effect of specific immunotherapy (SIT). Strong experimental evidence has been provided that these antibodies – mostly IgG – prevent the allergen-induced activation of mast cells and basophils by competing with IgE for the binding to allergen molecules [reviewed in 15, 86, 87].

Despite improved efficacy of extract-based immunotherapy, many patients still do not exhibit significant clinical improvement after as much as three years of therapy. In defining the reasons for the failure of SIT, several factors must be considered. First, in the case of complex allergen sources like timothy grass pollen or house dust mite that contain multiple allergens that can trigger symptoms, sensitization profiles may vary significantly between allergic individuals [88]. Extract-based measurement of IgG after immunotherapy does not provide information that the vaccination has induced IgG to all allergen molecules that the patient is sensitized to, as only total reactivity to an allergen source is measured when extracts are used. In addition, since extract-based tests contain both allergens and non-allergenic components, the serological test may also be positive in the case of exclusive production of therapeutically irrelevant IgG to non-allergenic components.

These diagnostic constraints have been partially overcome with the availability of serological tests comprising purified allergen molecules [89], as only antibodies specific to defined allergens are detected by these tests. Generally, two types of tests are available: tests for antibodies specific to single allergen molecules, and test systems using microarray technology that measure Ig reactivities to more than 100 components in a single step [90, 91••, 92].

In tests for reactivity to single allergen molecules, a relatively large amount of the purified allergen molecule is used for Ig detection when compared to average titers of allergen-specific antibodies in the serum. Hence, all antibodies in the sample that recognize the respective allergen – IgE as well as IgG – can be bound and detected. In this way, the test allows quantitative measurement of the respective antibody species, which may be relevant, for example, in the quantification of an IgE-boost by vaccination. However, if the IgG-response to SIT has to be measured, results of this test may be misleading because they do not distinguish blocking from non-blocking IgG. Even if only therapeutically irrelevant non-blocking IgG were induced, the test would yield a positive result. Furthermore, when assessing the immunological efficacy of SIT, it is important to determine that a vaccination produced a protective IgG “shield” covering all relevant allergens the patient is sensitized to. In the case of complex allergen sources, many single tests are required to obtain a comprehensive analysis of the patient’s immune response.

Allergen microarrays [90] typically comprise a large number of allergen molecules, usually more than 100 components, yielding the patient’s Ig-reactivity profile in a single step. With this technology, it is possible to assess whether a vaccination with an allergen extract successfully induced IgG to all relevant allergen components, or whether, due to the lack of a particular allergen in the extract, for example, IgG-reactivity to the allergen could not be achieved, which would thus explain therapeutic failure. Likewise, the blocking ability of allergen-specific IgG can be determined by microarray [91••]. In this instance, because only small amounts of protein are coupled to the surface (in the range of 50–200 fg per spot, which is approximately 10,000,000 times less than in tests comprising single allergens), this results in the restriction of the number of epitopes available for antibody binding. If a sample contains both IgE and IgG that bind to the same allergen but to different epitopes, both isotypes can bind independently without affecting the binding of the other isotype. By contrast, if IgE and IgG bind to the same epitopes or to epitopes in close proximity on the allergen surface, competition will mutually decrease the detected IgE and IgG levels. Therefore, a decreased IgE signal detected by microarray after specific immunotherapy as compared to the level before SIT may indicate that blocking IgG antibodies have been induced.

In this context, allergen microarrays facilitate analysis of the immune response of a patient treated by specific immunotherapy, enabling the physician to identify “holes” in the protective IgG-shield and to distinguish clinically relevant from insufficient IgG responses upon vaccination.

In addition to these clinically well-established serological tests, several methods are under investigation that may help assess and predict the success of SIT [93] or distinguish clinically beneficial from adverse immune responses to SIT [94]. Assays like the basophil activation test, for example, could serve as surrogates for *in vivo* tests [95], while the IgE-FAB assay allows the determination the blocking effect of SIT on IgE-facilitated allergen presentation [96].

## Personalized allergy treatment based on allergen components: closer than we think?

The goal of personalized medicine is to tailor and/or optimize treatment for each individual patient exactly to his or her personal situation. The topic has been widely discussed in the context of genetics, and largely advocates the use of new technologies to customize medicine for the benefit of patients. It may be considered ironic that customized medicine prescribed by medical doctors for individual patients and manually compounded by pharmacists was far more common before production and packaging of medicine became widely industrialized. Along the same lines, conventional allergen immunotherapy could also be regarded as a classic form of personalized medicine. In Europe, therapeutic allergen extract preparations were and still are largely ordered from pharmaceutical companies that produce, package, and label them for individual patients.

Similar to other pre-packaged medicines, few immunotherapy preparations have received full regulatory approval in the EU. Although the regulatory situation varies among different countries, drug products customized for individual patients generally do not have the same stringent approval requirements as other drugs [97]. On this basis, it was possible to make available a multitude of allergen extracts from a wide variety of allergen sources for the treatment of allergic patients, and the efficacy of many of these extracts has never been proven in controlled clinical trials. However, the widespread use of component-resolved diagnosis has revealed that most patients are sensitized to only a small number of different proteins. It has become clear that due to the high degree of cross-reactivity between allergen extracts from different sources, most allergic patients could be effectively treated with a small number of well-selected allergen components. This would have several advantages, including the avoidance of injecting allergens against which patients have no IgE sensitization.

In contrast to allergen extracts, market authorization of vaccines based on allergen components, recombinant allergens, allergen derivatives, and allergen peptides is extremely stringent and closely regulated (European Medicines Agency (EMA) guidelines published June 1, 2009: http://www.ema.europa.eu/docs/en_GB/document_library/Scientific_guideline/2009/09/WC500003605.pdf ). At this time, allergen vaccines need to undergo tests for market authorization in their final composition. This means that current regulations represent a hurdle for obtaining market authorization for individual components for use in different mixtures according to sensitization profiles of patients. As clinical trials for immunotherapy preparations are extremely costly, the current regulatory situation certainly slows the development of personalized treatment for allergic patients based on individual sensitization patterns. One way to speed up progress and improve the situation would be to allow market authorization for combination vaccines based on studies demonstrating common immunological mechanisms for individual allergen components, as is common practice in the field of vaccines for infectious diseases.

In summary, therefore, although it is already technically possible to treat each individual allergic patient precisely according to his or her sensitization profile, this practice is currently held back by the prevailing market authorization guidelines. Clinical studies investigating common mechanisms of SIT for different defined allergen components are urgently needed as the basis for new guidelines to allow the development of personalized forms of SIT.

## Outlook – prophylactic vaccination against allergies

Allergen-specific immunotherapy (SIT) is the only causative and disease-modifying treatment for IgE-mediated allergies. However, SIT aims to change an already established and misled immune response. The treatment does not cure the disease or restore healthy immune response [86]. Because allergies are a major health problem with high socioeconomic costs, it would be of great interest to have a prophylactic form of treatment that can prevent the development of IgE-mediated allergies, as has been standard for many years in infectious disease (e.g., hepatitis B, tetanus, or poliomyelitis).

There is no reason to believe that a prophylactic vaccination against IgE-mediated allergies is not possible, but it would have to fulfil certain requirements. First, and most importantly, the vaccine must be safe. Current SIT preparations are based on natural allergen extracts that can lead to an increase of allergen-specific IgE in allergic patients, and which would therefore bear the risk of sensitizing non-allergic individuals when used as prophylactic treatment [98]. It is possible that this issue could be overcome by the use of suitable hypoallergenic vaccines.

**Figure 1:**
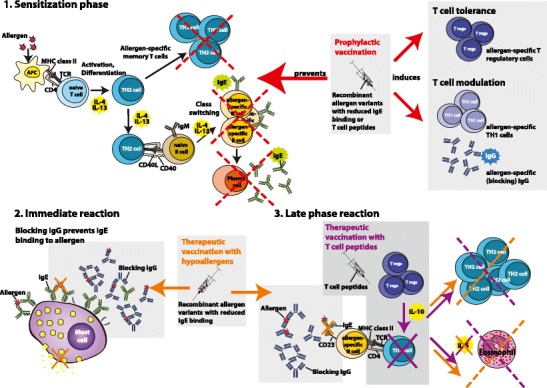
Graphical depiction of the immunological effects of a prophylactic allergy vaccine on the sensitization phase (1) and of therapeutic vaccination with hypoallergens and T cell peptides on immediate- (2) and late-phase (3) reactions.

In this context, recombinant allergen variants lacking the binding sites for allergen-specific IgE would be good candidate molecules for prophylactic vaccination due to the very low risk of sensitization to naturally occurring allergens. The immunological effect of this form of treatment is thought to be the induction of protective blocking IgG antibodies, similar to prophylactic vaccines against infectious diseases. These allergen-specific antibodies should bind to the allergen and thus prevent stimulation of the immune system (Fig. 1). Another possible approach is the application of allergen-specific T cell peptides with the goal of inducing tolerance (Fig. 1) (reviewed in [99•]).

A second very important aspect for a prophylactic treatment is the selection of the best time window for vaccination. It is the nature of a prophylactic treatment that it must be performed before the onset of the disease. The investigation of the precise time when sensitization occurs is currently the subject of large birth cohort studies [100] (http://medall-fp7.eu). It is thought that individuals with an atopic genetic background are sensitized in the first year(s) of life and become symptomatic when a certain threshold of allergen-specific IgE is reached [101]. After this primary sensitization phase, the IgE reactivity profile of allergic adults does not appear to undergo further relevant change [102]. In this context, the scenarios for prophylactic allergy vaccination under discussion include active vaccination or passive application of blocking antibodies to the pregnant allergic mother and/ or early postnatal active vaccination of the offspring with the goal to induce tolerance. Several animal models have demonstrated that the transmission of allergen-specific protective IgG antibodies via the placenta or breast milk can prevent allergic sensitization of the offspring [103, 104]. Furthermore, there is evidence that SIT performed in pregnant mothers may prevent allergic sensitization of the child as SIT-induced blocking IgG are transferred through the placenta [105, 106].

The third aspect that must be considered is the selection of the most useful panel of allergens. The definition of the most important allergens (i.e., those which most frequently cause allergic symptoms and those which cause more severe forms of disease) is currently the subject of investigations delineating sensitization profiles in different countries and populations [100, 107•] (http://medall-fp7.eu).

And, lastly, a target population must be defined of children who would benefit most from a prophylactic treatment and should therefore be vaccinated. Depending on the type of vaccine, it is conceivable that certain risk groups (e.g., those with an atopic family background) should be treated. An initial attempt to further pursue this approach is currently undergoing clinical investigation. Two hypoallergenic fragments of Bet v 1 that have previously been shown to induce protective immune responses but no new IgE antibody responses in birch pollen-allergic patients are currently being used for vaccination of 20 non-allergic adult individuals in a clinical phase I study (NCT01353924).

## Summary

Over the last 25 years, most of the clinically relevant allergens from important allergen sources have been isolated and produced as recombinant molecules. Allergen components are already used in routine diagnostic setting, but they can also improve the monitoring of patients undergoing allergen immunotherapy. Panels of recombinant allergens may also replace natural allergen extracts for the treatment of allergic patients. Genetic engineering allows the production of innovative allergy vaccines designed to reduce side effects, enhance clinical efficacy, and increase treatment convenience. Based on several clinical studies performed with recombinant allergens, hypoallergens, and peptides, it is clear that such vaccines will greatly improve SIT and may be useful for prophylaxis. However, further clinical studies are needed for these new vaccines to become available for patients.
